# Bariatric Surgery for Monogenic Non-syndromic and Syndromic Obesity Disorders

**DOI:** 10.1007/s11892-020-01327-7

**Published:** 2020-07-30

**Authors:** Niels Vos, Sabrina M. Oussaada, Mellody I. Cooiman, Lotte Kleinendorst, Kasper W. ter Horst, Eric J. Hazebroek, Johannes A. Romijn, Mireille J. Serlie, Marcel M. A. M. Mannens, Mieke M. van Haelst

**Affiliations:** 1 Department of Clinical Genetics, Amsterdam UMC, University of Amsterdam, Amsterdam Reproduction and Development Research Institute, Meibergdreef 9, Amsterdam, The Netherlands; 2 Department of Endocrinology and Metabolism, Amsterdam UMC, University of Amsterdam, Meibergdreef 9, Amsterdam, The Netherlands; 3 Department of Bariatric Surgery, Rijnstate Hospital and Vitalys Clinic, Arnhem, The Netherlands; 4 Department of Internal Medicine, Amsterdam UMC, University of Amsterdam, Meibergdreef 9, Amsterdam, The Netherlands; 5 Genome Diagnostics Laboratory, Department of Clinical Genetics, Amsterdam UMC, University of Amsterdam, Amsterdam Reproduction and Development Research Institute, Meibergdreef 9, Amsterdam, The Netherlands; 6 Department of Clinical Genetics, Amsterdam UMC, Vrije Universiteit Amsterdam, Boelelaan 1117, Amsterdam, The Netherlands

**Keywords:** Obesity, Genetics, *MC4R*, Bariatric surgery, PWS

## Abstract

**Purpose of Review:**

The global prevalence of obesity has increased rapidly over the last decades, posing a severe threat to human health. Currently, bariatric surgery is the most effective therapy for patients with morbid obesity. It is unknown whether this treatment is also suitable for patients with obesity due to a confirmed genetic defect (genetic obesity disorders). Therefore, this review aims to elucidate the role of bariatric surgery in the treatment of genetic obesity.

**Recent Findings:**

In monogenic non-syndromic obesity, an underlying genetic defect seems to be the most important factor determining the efficacy of bariatric surgery. In syndromic obesity, bariatric surgery result data are scarce, and even though some promising follow-up results have been reported, caution is required as patients with more severe behavioral and developmental disorders might have poorer outcomes.

**Summary:**

There is limited evidence in support of bariatric surgery as a treatment option for genetic obesity disorders; hence, no strong statements can be made regarding the efficacy and safety of these procedures for these patients. However, considering that patients with genetic obesity often present with life-threatening obesity-related comorbidities, we believe that bariatric surgery could be considered a last-resort treatment option in selected patients.

**Electronic supplementary material:**

The online version of this article (10.1007/s11892-020-01327-7) contains supplementary material, which is available to authorized users.

## Introduction

Obesity is one of the leading health problems globally. According to the World Health Organization (WHO), the global number of patients suffering from obesity has almost tripled over the past 45 years, resulting in an increased prevalence of obesity-associated comorbidities, including cardiovascular diseases and diabetes. These comorbidities are the most common causes of death worldwide [1, 2]. To maintain a healthy weight, a balance between energy intake and energy expenditure is required [3]. An energy imbalance, with a relatively larger energy intake than expenditure, can lead to obesity, which is defined as a body mass index (BMI) ≥ 30 kg/m^2^ for adults or ≥ 2 or 3 standard deviations (SD) for children over or under the age of 5 years old, respectively [2]. Obesity is a complex multifactorial disorder caused by the interplay of environmental and genetic factors [4, 5]. An underlying genetic cause can be identified in 5–10% of patients with severe and/or early-onset obesity [5–10]. A defect in one (monogenic) or multiple genes (polygenic) can result in obesity. This can be isolated (non-syndromic obesity) or in a more complex clinical presentation, where apart from obesity, other organ systems are affected, with or without intellectual deficit (syndromic obesity). Apart from monogenic and polygenic causes, there can also be epigenetic changes that play a role in the pathogenesis of obesity, as has been recently described by Rohde et al. [11].

In general, weight loss is achieved by changing the energy balance to a point where energy expenditure exceeds intake. Initial therapeutic options focus on lifestyle interventions aimed at reducing dietary intake, increasing physical activity, treating underlying pathology, and/or pharmacological strategies. However, when conservative therapeutic options fail to result in sustainable weight loss and morbid or severe obesity with associated comorbidities persists (respectively, BMI > 40 kg/m^2^ and BMI > 35 kg/m^2^ in adults), bariatric surgery becomes a viable treatment option.

Bariatric surgery is the most effective treatment for patients with severe or morbid obesity [12]. Nowadays, the most performed surgical techniques include sleeve gastrectomy (SG) and Roux-en-Y gastric bypass (RYGB). Adjustable gastric banding (AGB), a technique widely performed in the past, showed disappointing results in the long term and is as such no longer a standard procedure [13]. Besides reducing stomach volume and altering food passage through the intestines, the neuroendocrine and gut-brain axis alterations are thought to play a significant role in the weight loss response [14–16].

Although most patients experience favorable effects of bariatric surgery, inter-individual variability in surgical outcomes is observed [17]. Several etiological factors, such as differences in the underlying pathophysiology of obesity can cause insufficient weight loss or weight regain. Concerning monogenic non-syndromic and syndromic obesity, it is hypothesized that some patients might not benefit from certain types of bariatric surgery when compared with patients with non-genetic obesity. This review provides an overview of the current knowledge of bariatric surgery outcomes in patients with a proven genetic form of obesity and aims to provide recommendations concerning the most suitable treatments in various genetic obesity disorders.

## Bariatric Surgery for the Treatment of Non-syndromic Genetic Obesity

**Fig. 1:**
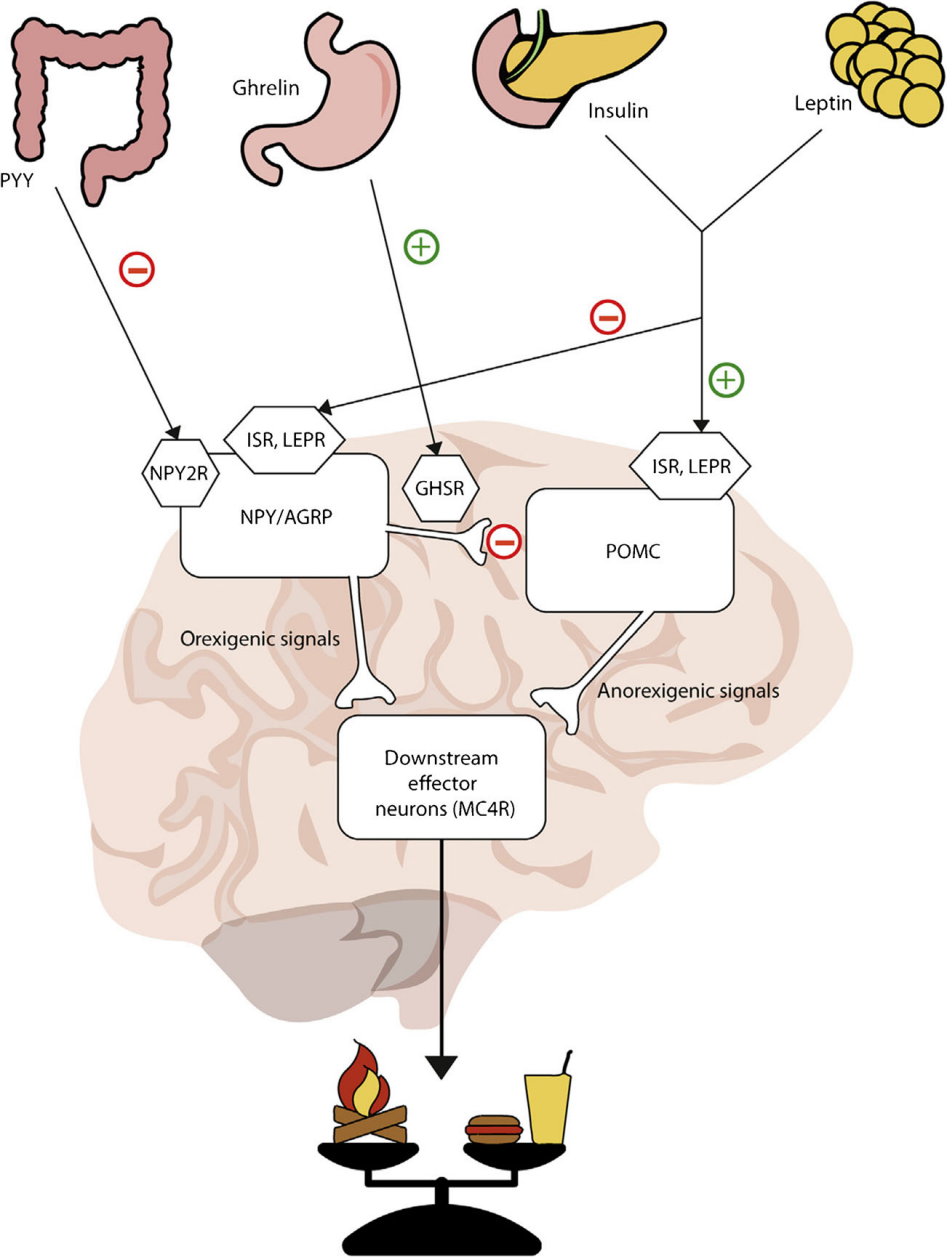
The leptin-melanocortin pathway and its effect on energy balance. Reprinted from the *Clinical Molecular Medicine: Principles and Practice* (p.80), by L. Kleinendorst and M. M. van Haelst, published by Elsevier, Copyright © 2020 by Elsevier Inc. Reprinted with permission [22]

Non-syndromic genetic obesity is usually the result of a defect in the leptin-melanocortin pathway (Fig. 1). This pathway plays an essential role in energy homeostasis. Signals from peripheral tissues are processed in the hypothalamus, regulating food intake and thus affecting body weight. Most genes involved in this pathway have been extensively studied and include *MC4R*, *LEPR*, *POMC*, *PCSK1*, and *SIM1* [18–21].

### Melanocortin-4-Receptor

The most frequent cause of non-syndromic genetic obesity is a mutation in the melanocortin-4-receptor (*MC4R*) gene. Mutations in this gene typically lead to a defect of the melanocortin-4-receptor, resulting in a phenotype of early-onset severe obesity with hyperphagia [23, 24]. Carriers of a single *MC4R* mutation are typically less severely affected than patients with compound heterozygous or homozygous mutations [25]. Bariatric surgery outcomes of patients with obesity resulting from *MC4R* gene defects (heterozygous, compound heterozygous, and homozygous mutations) have been described.

A 3-year follow-up study showed poor results of AGB in nineteen patients with *MC4R* mutations. There was 18% less total weight loss in carriers of an *MC4R* mutation, compared with non-carriers (*p* = 0.003). In addition, there were more gastric complications (mean 0.303 ± 0.060 SEM versus 0.057 ± 0.010 SEM; *p* < 0.001) and reoperations (57.9% versus 14.2%, *p* ≤ 0.001) in the *MC4R* mutation carriers, compared with non-carriers. However, when reoperation with RYGB occurred, weight loss was similar between the two groups. In this specific cohort, a higher frequency of binge eating disorder (BED) was observed in patients with *MC4R* mutations (100% versus 18.1%; *p* < 0.001), which is thought to explain, at least in part, the difference in outcome after AGB [26]. In six other studies, no differences in outcomes were seen 1 year after AGB or RYGB [27–29], 1 to 5 years after AGB or SG [30, 31], and 84 months after RYGB [32] between heterozygous *MC4R* mutation carriers compared with non-carriers. Furthermore, a case report with long-term follow-up results showed beneficial effects of RYGB in one patient with an *MC4R* mutation (76% excess weight loss after almost 5 years of follow-up) [33].

Homozygous loss-of-function (LOF) mutations in *MC4R* have been described in four pediatric patients who underwent SG. While initial results were promising in all patients, one patient had weight regain after 5 years of follow-up. Nonetheless, the authors state that SG is a suitable option for pediatric patients with severe obesity and homozygous *MC4R* mutations [34•].

In a large recent Swiss study, *MC4R* analysis was performed in 872 patients who received bariatric surgery, revealing fourteen *MC4R* gene variants. Of these variants, eleven were previously described: nine LOF (of which two were found in homozygous form) and two gain-of-function (GOF) variants. Surgical techniques used in this cohort were AGB, RYGB, or a combination of the two (hybrid; HYB). Patients with *MC4R* variants showed poorer outcomes, with more complications and reoperations after bariatric surgery. This is potentially attributable to the AGB component. However, weight loss was similar (BMI loss 13–14 kg/m^2^ on average in *MC4R* variant carriers versus 12 kg/m^2^ in non-carriers, percentages not shown). The authors of this study recommend *MC4R* analysis before surgery in order to choose the right type of surgery [35•]. The reported *MC4R* gene variants in this study were, however, not all (likely) pathogenic, making it hard to draw firm conclusions.

In our recently reported large bariatric surgery cohort, consisting of 1014 patients, an *MC4R* mutation was found in 1%. No differences in outcomes between *MC4R* mutation carriers and non-carriers were observed after RYGB after up to 2 years of follow-up. However, SG was less effective in *MC4R* mutation carriers compared with the rest of the cohort. Therefore, RYGB might be more suitable for patients with an *MC4R* mutation than SG [36••].

### Leptin Receptor

Another important gene in this leptin-melanocortin pathway is the leptin receptor (*LEPR)* gene. Biallelic genetic defects in this gene are rare, and only 88 patients with a homozygous or compound heterozygous *LEPR* gene mutation have thus far been described in the literature. Patients with biallelic *LEPR* mutations typically show hyperphagia and early-onset obesity, among other endocrine disturbances [37]. Of those 88 patients, six have had bariatric surgery. Bariatric surgery was unsuccessful in the three females with a biallelic *LEPR* mutation; they all regained their lost weight at follow-up. Two female patients underwent RYGB; one lost 50 kg (22.7% of total body weight) but regained it all during pregnancy [38], and the other had a 45 kg weight loss (27.8% of total body weight) after 17 months but regained 34 kg weight 5 years after surgery [39]. The third female patient received SG. She lost 30 kg and regained 19 kg (% of total weight not reported) within an unknown time frame [40••]. The three males with biallelic *LEPR* mutations that underwent bariatric surgery all showed good results. The first patient had a weight loss of 46 kg (28%) a year after AGB and regained it after slippage of the band. A second gastroplasty resulted in a weight loss of 20%, which he maintained after 8 years of follow-up [41]. The second male patient was a 14-year-old boy who lost 47 kg after AGB and regained 10 kg after slippage of the band. His BMI changed from 53.7 preoperatively to 41.6 kg/m^2^ (− 22.5%) after a follow-up period of 15 years [40••]. The third male patient had a 44% weight loss**,** 9 months after gastroplasty, but no long-term follow-up data were reported [39]. It is interesting to see a sex-specific difference in outcomes in patients carrying biallelic *LEPR* mutations. Sex differences are more often described in patients with biallelic *LEPR* mutations [42]. However, no firm conclusions can be drawn on such a heterogeneous group, with only a small number of patients.

### Pro-opiomelanocortin

The pro-opiomelanocortin (*POMC*) gene is another well-studied gene in the leptin-melanocortin pathway. POMC is cleaved into adrenocorticotropin (ACTH), β-endorphin, and multiple melanocyte-stimulating hormones (MSHs; α-MSH, β-MSH, and multiple γ-MSHs). These hormones are called melanocortins, and they bind to the melanocortin receptors (MCRs). Four different MCRs show different responses upon binding. MC1R stimulation affects skin and hair pigmentation, whereas MC2R activation results in cortisol production, and MC3R and MC4R (as described above) are important in the regulation of food intake and energy homeostasis [43–45]. Patients with biallelic *POMC* gene mutations present with a POMC deficiency syndrome, characterized by red hair, pale skin, adrenal insufficiency, and early-onset obesity. Heterozygous *POMC* mutations result in a susceptibility to childhood obesity [43, 46]. Little is known about the effectiveness of bariatric surgery in the treatment of obesity in patients with *POMC* mutations.

Two studies describe patients with *POMC* variants that undergo AGB or RYGB [26, 38]. In one of these studies, it is unlikely that the reported variants are pathogenic and bariatric surgery outcome results were not different from the rest of the cohort [26]. In our recent study, twelve patients with heterozygous pathogenic *POMC* variants responded well to SG and RYGB up to 2 years of follow-up [36••].

### Other Monogenic Forms of Obesity

Less is known about the other important components of the leptin-melanocortin pathway. These components include the single-minded homolog 1 (*SIM1*) and proprotein convertase subtilisin/kexin type 1 (*PCSK1*) genes.

*SIM1* encodes a transcription factor that is essential for the development of part of the hypothalamus (paraventricular, anterior periventricular, and supraoptic nuclei). A defective *SIM1* gene is associated with hyperphagia and severe early-onset obesity [47]. In our previous study, one patient with a heterozygous *SIM1* mutation is described with a revisional RYGB after initially receiving AGB. After 36 months, a weight loss of more than 20% was maintained. Five other patients were carriers of single variants of unknown significance in *SIM1*, of which one has recently been reclassified to likely pathogenic after functional assessment [36, 48]. This patient had a 28.8% total body weight loss after 2 years of follow-up after RYGB.

*PCSK1* is responsible for the synthesis of prohormone convertase 1/3 (PC1/3). This convertase is important for the cutting of proteins, like POMC (resulting in the production of melanocortins) [49]. Defects in this gene result in a broad phenotype, including obesity, endocrine manifestations (hypogonadotropic hypogonadism, changes in adrenal and thyroid functioning, poor regulation of blood glucose), and malabsorptive diarrhea [50]. Our previous study described five patients with pathogenic *PCSK1* mutations, who underwent RYGB, resulting in a total weight loss of over 20% for all three patients at 1- to 3-year follow-up. No differences in outcomes after the different procedures were observed compared with the rest of our cohort [36••]. At present, there are no other published studies that clearly describe the outcomes of bariatric surgery in individuals with a confirmed molecular cause of non-syndromic obesity.

An overview of the studies that were included in this review is provided in Table 1.

**Table 1:** Overview of the included articles (*n* = 23) on bariatric surgery outcomes in patients with genetic obesity

Author, year	Total cohort (*n*)	Genetic defect/disorder	Genetic obesity, (*n*)*	Surgical technique	Follow-up (years)	Results at follow-up
Non-syndromic						
Nunziata et al. (2019), review	57	Biallelic *LEPR* mutations	6	AGB, RYGB, SG, gastroplasty	0.75–15	Long-lasting weight loss in males (*n* = 3). Weight regain in females (*n* = 3)
Huvenne et al. (2015)	12	Biallelic *LEPR* mutations	2	Gastric bypass, gastroplasty	?–15	See Nunziata et al. (2019)
Le Beyec et al. (2013)	1	Biallelic *LEPR* mutations	1	Gastric banding, gastroplasty	8	*See* Nunziata et al. (2019)
Nizard et al. (2012)	1	Biallelic *LEPR* mutations	1	Abdominoplasty, gastric bypass	2	See Nunziata et al. (2019)
Mul et al. (2012)	46	*MC4R*	5	SG	1	No difference in outcome
Bonnefond et al. (2016)	872	*MC4R*	64**	AGB, RYGB, Hybrid	6	Significantly worse outcomes and higher rate of BED and LOC in *MC4R* variant** carriers, compared with non-carriers. Variants were GOF (*n* = 47) and LOF (*n* = 17)
Jelin et al. (2016)	4	*MC4R* deficiency	4	SG	0.4–5	Significant short-term weight loss in all patients. Long-term weight regain in one patient. Children
Elkhenini et al. (2014)	1	*MC4R*	1	RYGB	5	76% EWL
Moore et al. (2014)	1433	*MC4R*	18**	RYGB	7	Similar weight loss for *MC4R* variant** carriers compared with controls
Censani et al. (2014)	135	*MC4R*	4	AGB, SG	1–5	36–85% EWL after AGB in three patients. 96% EWL after SG in one patient
Hatoum et al. (2012)	972	*MC4R*	15	RYGB	3	Similar weight loss in patients with *MC4R* mutations, compared with non-carriers
Valette et al. (2012)	648	*MC4R*	9	AGB, RYGB	1	No difference in weight loss
Aslan et al. (2011)	92	*MC4R*	4	RYGB	1	66% EWL compared with 70% EWL in controls
Cooiman et al. (2020)	1014	*MC4R*, *PCSK1*, *POMC*, *SIM1*	30	RYGB, SG	2	*MC4R* (*n* = 11): RYGB more effective than SG
						*PCSK1* (*n* = 5)*, POMC* (*n* = 12), and *SIM1* (*n* = 2, including one likely pathogenic variant): no significant differences
Potoczna et al. (2004)	300	*MC4R*, *POMC, LEPR*	?**	AGB, RYGB (reoperation)	3	*MC4R* variant** carriers (*n* = 19) versus controls: 18% less TWL (*p* = 0.003), higher reoperation rate (57.9% versus 14.2%), and more BED (100% versus 18.1%). *POMC* variant** carriers (*n* = 144) and *LEPR* variant** carriers (*n* = 247) versus controls: no difference in outcomes
Syndromic						
Ferrario et al. (2014)	1	AHO (PPHP)	1	SG	3	46.8% TWL
Ates et al. (2018)	8	BBS	1	SG	1	28% TWL. Child
Boscolo et al. (2017)	1	BBS	1	SG	3	32.6% TWL
Martinelli et al. (2019)	1	PWS	1	SG	0.5	29.2% EWL. Child
Cazzo et al. (2018)	1	PWS	1	BPD	1	55% EWL
Alqahtani et al. (2016)	96	PWS	24	SG	3	Similar BMI loss after SG compared with controls. Children
Michalik et al. (2015)	2	PWS	2	BPD	0.5–1.5	EWL of 43% (*n* = 1 at 6 months). EWL of 25% (*n* = 1 at 18 months)
Scheimann et al. (2008), review	60	PWS	60	BPD, intragastric balloon, gastric bypass, other	2	Mixed results; weight loss of 0 to 40%, weight gain up to 2%, depending on technique, small number of patients

*AGB* adjustable gastric banding, *AHO* Albright hereditary osteodystrophy, *BBS* Bardet-Biedl syndrome, *BED* binge eating disorder, *BPD* biliopancreatic diversion, *EWL* excess weight loss, *GOF* gain of function, *Hybrid* combination AGB and RYGB, *LOF* loss of function, *PPHP* pseudopseudohypoparathyroidism, *PWS* Prader-Willi syndrome, *RYGB* Roux-en-Y gastric bypass, *SG* sleeve gastrectomy, *TWL* total weight loss

*Number of patients with genetic defects who underwent bariatric surgery

**Genetic defects of varying pathogenicity

## Bariatric Surgery for the Treatment of Syndromic Obesity

Syndromic obesity is defined as adiposity combined with dysmorphic features, organ-specific congenital anomalies, and/or developmental delay/intellectual disability. Currently, more than 79 obesity syndromes have been described [51], of which Prader-Willi and Bardet-Biedl syndromes are most frequently reported [3]. In addition to general weight loss measures, such as lifestyle interventions, cognitive behavioral therapy, or pharmacotherapy, there are no specific evidence-based therapies for syndromic obesity. Here, we provide an overview of the available evidence on the various bariatric procedures for the treatment of three (monogenic) obesity syndromes (see Table 1). We note that data are limited, since the use of such invasive treatments in patients with genetic obesity syndromes remains controversial, and that there is no international consensus as yet.

### Prader-Willi Syndrome

In most cases, Prader-Willi syndrome (PWS) is caused by loss or disruption of the paternal copy of chromosome 15q11.2-13 and has an estimated prevalence of 1:8000–1:50,000 individuals [52, 53]. PWS is characterized by hypotonia, feeding difficulties, and mild dysmorphic features in the neonatal period. As patients become older, a global developmental delay, neuropsychiatric and endocrine manifestations (short stature, hypogonadism, hypothyroidism, adrenal insufficiency), and more distinctive dysmorphic features can be observed. Feeding difficulties typically dissolve after the age of 9 months, and weight increases more rapidly after the age of 2. From around the age of 4 onwards, food intake increases, and excessive eating (hyperphagia) can be observed [54, 55]. This usually results in childhood-onset morbid obesity and its associated complications (obstructive sleep apnea, diabetes mellitus type 2, and hypertension). In fact, the associated complications of their insatiable appetite and uncontrolled weight gain are the leading causes of death during adolescence or early adulthood [56, 57].

Current obesity treatments that focus on changes in feeding behavior/diet, exercise, or hormonal replacement therapy are ineffective for long-term weight loss maintenance in the majority of patients with PWS [58]. Although bariatric procedures, including RYGB, result in long-lasting weight reduction and remission of comorbidities in the majority of patients with obesity in the general population [59], it remains controversial whether or not to perform this invasive therapy in patients with PWS. The pathophysiology of obesity in PWS is different from other forms of genetic obesity; the compulsive food seeking and behavioral problems in PWS are thought to interfere with lifestyle changes needed after bariatric surgery. Previously reported bariatric procedures, including intragastric balloon placement or gastric banding, did not produce favorable weight loss outcomes in patients with PWS [60].

Today’s first-choice weight loss procedures (SG and RYGB) have been studied in one cohort of patients with PWS. SG was performed in 24 pediatric patients with molecularly confirmed PWS, and outcomes were compared with the outcomes of 72 children with obesity without PWS, who were matched for age, gender, and BMI at baseline [61•]. Children in the study were 4.9–18.2 years old. After surgery, children with PWS lost considerable body weight, and there were no apparent differences in postoperative BMI loss between the PWS and control groups up to 3 years after sleeve gastrectomy. However, in the 4th and 5th year of follow-up, the PWS group regained weight, which resulted in a mean (±SD) BMI of 35.9 ± 12.5 kg/m^2^, compared with 25.1 ± 7.0 kg/m^2^ in the control group. This finding suggests that patients with PWS may be more prone to long-term weight regain, but more (complete) data are needed to confirm this trend. No short- or long-term complications were reported.

In addition to this case-control study, there are four single PWS cases reported for which outcomes of sleeve gastrectomy or biliopancreatic diversion were reported. A 25-year-old man with PWS, with a preoperative BMI of 55 kg/m^2^ and impaired glucose tolerance (IGT), underwent an uncomplicated biliopancreatic diversion [62••]. One year after surgery, his BMI had decreased to 38.5 kg/m^2^ (− 30%), and his IGT had resolved. A 16-year-old adolescent with PWS and a preoperative BMI of 80.9 kg/m^2^ also suffered from severe obstructive sleep apnea with nocturnal respiratory failure, hypertension, and IGT [63••] and had a reduction in BMI to 64.6 kg/m^2^ (− 20.1%) 6 months after SG. Finally, two PWS patients were reported who underwent a biliopancreatic diversion [64•]. The first case was a 25-year-old woman with a decrease in BMI from 55.5 to 41.3 kg/m^2^ after 6 months, corresponding to an excess weight loss (EWL) of 43%. The second case, an 18-year-old woman, with a preoperative BMI of 64.4 kg/m^2^, showed a decrease to 53.9 kg/m^2^ (− 16.3%) within 18 months after surgery. Notably, prior treatment with an intragastric balloon had failed to achieve weight loss.

Based on the available observational data, it seems that short-term bariatric surgery-induced weight loss outcomes in patients with PWS are comparable with those achieved in other patients with (non-genetic) obesity. However, these limited data do not (yet) justify the widespread application of bariatric surgery in patients with PWS, as gastric rupture and necrosis have been previously described in patients that did not even undergo bariatric surgery [65]. Long-term outcome needs to be further studied for these patients. Intensive postoperative behavioral therapy and supervision might prevent or delay weight regain, so that bariatric surgery for patients with PWS might reduce obesity-associated morbidity and mortality.

### Bardet-Biedl Syndrome

Bardet-Biedl syndrome (BBS) is a rare autosomal recessive ciliopathy. BBS is characterized by severe early-onset obesity, intellectual deficit, polydactyly, renal abnormalities, and retinitis pigmentosa [66]. Although more than 20 genes are currently known to be associated with BBS, mutations in *BBS1* and *BBS10* are identified in the majority of cases [66, 67]. Weight loss strategies focusing on lifestyle changes and pharmacotherapy do not seem to result in sustained weight loss in affected patients [68, 69]. Therefore, other therapies, including bariatric surgery, are being explored. During the last 5 years, two case reports of SG in patients with BBS have been published.

A 37-year-old woman with BBS who had morbid obesity underwent uncomplicated SG [70••]. Her BMI decreased from 40.8 to 27.5 kg/m^2^ (− 32.6%) within 36 months after surgery, with additional improvements in glycemic control, hypertension, and non-alcoholic fatty liver disease. Another study examined the effects of SG in adolescents, including one person who was diagnosed with BBS [71••]. The postoperative period of the 14-year-old boy was uneventful. The intervention was associated with a total weight reduction of 28% after 12 months. His blood pressure normalized, and hypertension treatment could be discontinued.

Thus, although the available evidence on modern bariatric surgery techniques in patients with BBS is scarce, the described cases suggest that SG may be a safe and effective treatment for BBS-related obesity, although more studies are needed to support the effectiveness in the long term.

### Albright Hereditary Osteodystrophy

Albright hereditary osteodystrophy (AHO) is an obesity syndrome with a broad spectrum of manifestations, including short stature, brachydactyly, and subcutaneous calcifications due to resistance to parathyroid hormone (pseudohypoparathyroidism; PHP) [72]. AHO is caused by heterozygous inactivation of the *GNAS* gene, encoding the alpha chain of the stimulatory G protein [73]. Genomic imprinting induces a variable phenotypic expression [74]. Maternal *GNAS* inactivation results in the AHO phenotype plus resistance to other hormones, most notably thyroid-stimulating hormone [74]. A loss of expression of *GNAS* of the paternal allele results in the AHO phenotype without hormonal resistance (pseudopseudohypoparathyroidism; PPHP). To our knowledge, no data are available regarding the effects of conservative weight-loss strategies in patients with AHO. However, there is one reported case of bariatric surgery in a patient with AHO.

A 26-year-old woman with PPHP (heterozygous mutation of the paternal *GNAS* gene), obesity, and type 2 diabetes underwent uncomplicated RYGB [75]. Within the first year after surgery, her BMI decreased from 49.5 to 25.9 kg/m^2^ (− 47.7%). Her body weight remained stable during the following 24 months. The effects of this weight loss on glycemic control were not discussed.

## Discussion

In summary (see Table 1), there is limited high-quality evidence in support of bariatric surgery as a treatment option for genetic obesity. In monogenic non-syndromic obesity, the identification of an *MC4R* mutation seems to be no contraindication for bariatric surgery. RYGB might be more suitable than SG for patients with an *MC4R* mutation. In patients carrying biallelic *LEPR* mutations, there seems to be a sex-specific effect, where males show better bariatric surgery outcomes. This is however based on only six patients in total. For the other monogenic non-syndromic obesity disorders, bariatric surgery outcome results seem to be similar to those of patients without an identified genetic cause of their obesity, but sample sizes were even smaller. The underlying mechanisms of genetic obesity syndromes are mostly unknown and might involve genetically determined dysregulations of neuronal circuits involved in the control of feeding behavior. Bariatric surgery is not only a restrictive and malabsorptive procedure but also affects the neuroendocrine milieu and the gut-brain axis [14–16]. The positive effect of the procedure on food intake could provide further insight into the neuronal regulation of feeding behavior in patients with these syndromes.

Limitations of the described studies are that some include genetic variants for which the pathogenicity is not certain or for which it is not sure that heterozygosity is the explanation of these patients’ obesity. A higher preoperative BMI is commonly observed in patients with a genetic obesity disorder, making it essential to compare the percentage of total weight or BMI loss, instead of the absolute weight loss in kg, which is frequently done. The rapid development of technology can facilitate further genetic analyses. Cases now being reported as non-genetic could well harbor a (yet unknown) genetic defect that will be identified in the near future. We noticed that some of the studies included in this review describe the application of bariatric surgery in young children. Although the safety of bariatric surgery in children and adolescents has been established in various studies, ethical issues are still a subject of ongoing debate [76, 77].

It would be interesting to systematically assess the effects of bariatric surgery on genetic versus non-genetic obesity. A systematic analysis could function as a tool to predict the effectiveness of surgical interventions in specific genetic obesity disorders. Also, the current rapid growth in knowledge of molecular pathways underlying genetic obesity will provide us with novel insights into the pathophysiological mechanisms in the development of obesity. Finally, we believe that there is an essential role for polygenic and epigenetic factors in the pathophysiology of obesity. Future studies regarding these mechanisms can potentially further explain the difference in outcomes and guide us in the right direction for personalized treatments of specific obesity disorders.

## Conclusion

Altogether, no strong statements can be made regarding the efficacy and safety of bariatric surgery procedures in patients with genetic obesity disorders. Our opinion is that available evidence does not (yet) support the widespread application of invasive bariatric procedures for patients with genetic obesity. In addition, since patients with genetic obesity often present with life-threatening obesity-related comorbidities, we believe that bariatric surgery could be considered as a last-resort option in a selection of patients with genetic obesity disorders, after careful consideration by an experienced and multidisciplinary obesity team, including behavioral therapists familiar with hyperphagia and compulsive eating disorders.

## Electronic Supplementary Material

ESM 1 (DOCX 22 kb)
