# Supplementary material for: Bariatric Surgery for Monogenic Non-syndromic and Syndromic Obesity Disorders
Source: Curr Diab Rep. 2020 Jul 30;20(9):44. doi: 10.1007/s11892-020-01327-7 (PMC7391392; doi:10.1007/s11892-020-01327-7)
Supplement: Supplementary file 1 — (DOCX 22 kb) [file 11892_2020_1327_MOESM1_ESM.docx]

**Supplementary Table 1:** Overview of the included articles (n=23) on bariatric surgery outcomes in patients with genetic obesity

| **Author, year** | **Total cohort(n)** | **Genetic defect/ disorder** | **Genetic obesity, (n)*** | **Surgical technique** | **Follow-up (years)** | **Results at follow-up** |
| --- | --- | --- | --- | --- | --- | --- |
| *Non-syndromic* | | | | | | |
| Nunziata et al, 2019, **review** | 57 | *LEPR* deficiency | 6 | AGB, RYGB, SG, gastroplasty | 0.75-15 | Long lasting weight loss in males (n=3). Weight regain in females (n=3). |
| Huvenne et al, 2015 | 12 | *LEPR*  deficiency | 2 | Gastric bypass, gastroplasty | ?-15 | *See Nunziata et al 2019* |
| Le Beyec et al, 2013 | 1 | *LEPR*  deficiency | 1 | Gastric banding, gastroplasty | 8 | *See Nunziata et al 2019* |
| Nizard et al, 2012 | 1 | *LEPR*  deficiency | 1 | Abdominoplasty, gastric bypass | 2 | *See Nunziata et al 2019* |
| Mul et al, 2012 | 46 | *MC4R* | 5 | SG | 1 | No difference in outcome. |
| Bonnefond et al, 2016 | 872 | *MC4R* | 64** | AGB, RYGB, Hybrid | 6 | Significantly worse outcomes and higher rate of BED and LOC in *MC4R* variant** carriers, compared to non-carriers. Variants were GOF (n=47) and LOF (n=17). |
| Jelin et al, 2016 | 4 | *MC4R* deficiency | 4 | SG | 0.4-5 | Significant short term weight loss in all patients. Long term weight regain in one patient. Children. |
| Elkhenini et al, 2014 | 1 | *MC4R* | 1 | RYGB | 5 | 76% EWL. |
| Moore et al, 2014 | 1433 | *MC4R* | 18** | RYGB | 7 | Similar weight loss for *MC4R* variant** carriers compared to controls. |
| Censani et al, 2014 | 135 | *MC4R* | 4 | AGB, SG | 1-5 | 36-85% EWL after AGB in three patients. 96% EWL after SG in one patient. |
| Hatoum et al, 2012 | 972 | *MC4R* | 15 | RYGB | 3 | Similar weight loss in patients with *MC4R* mutations, compared to non-carriers. |
| Valette et al, 2012 | 648 | *MC4R* | 9 | AGB, RYGB | 1 | No difference in weight loss. |
| Aslan et al, 2011 | 92 | *MC4R* | 4 | RYGB | 1 | 66% EWL compared to 70% EWL in controls. |
| Cooiman et al, 2020 | 1014 | *MC4R, PCSK1, POMC, SIM1* | 30 | RYGB, SG | 2 | *MC4R* (n=11): RYGB more effective than SG.  *PCSK1* (n=5)*, POMC* (n=12) and *SIM1* (n=2, including one likely pathogenic variant): No significant differences. |
| Potoczna et al, 2004 | 300 | *MC4R*, *POMC, LEPR* | ?** | AGB, RYGB (reoperation) | 3 | *MC4R* variant** carriers (n=19) versus controls: 18% less TWL (p=0.003), higher reoperation rate (57.9% versus 14.2%) and more BED (100% versus 18.1%). *POMC* variant** carriers (n=144) and *LEPR* variant** carriers (n=247) versus controls: no difference in outcomes. |
| *Syndromic* | | | | | | |
| Ferrario et al, 2014 | 1 | AHO (PPHP) | 1 | SG | 3 | 46.8% TWL. |
| Ates et al, 2018 | 8 | BBS | 1 | SG | 1 | 28% TWL. Child. |
| Boscolo et al, 2017 | 1 | BBS | 1 | SG | 3 | 32.6% TWL. |
| Martinelli et al, 2019 | 1 | PWS | 1 | SG | 0.5 | 29.2% EWL. Child. |
| Cazzo et al, 2018 | 1 | PWS | 1 | BPD | 1 | 55% EWL. |
| Alqahtani et al, 2016 | 96 | PWS | 24 | SG | 3 | Similar BMI loss after SG compared to controls. Children. |
| Michalik et al, 2015 | 2 | PWS | 2 | BPD | 0.5-1.5 | EWL of 43% (n=1 at 6 months). EWL of 25% (n=1 at 18 months). |
| Scheimann et al, 2008, **review** | 60 | PWS | 60 | BPD, intragastric balloon, gastric bypass, other | 2 | Mixed results; weight loss of 0 to 40%, weight gain up to 2%, depending on technique, small number of patients. |

AGB = Adjustable Gastric Banding, AHO = Albright hereditary osteodystrophy, BBS = Bardet-Biedl syndrome, BED = Binge Eating Disorder, BPD = Biliopancreatic diversion, EWL = Excess weight loss, GOF = Gain of function, Hybrid = Combination AGB and RYGB, LOF= Loss of function, PPHP = Pseudopseudohypoparathyroidism, PWS = Prader-Willi syndrome, RYGB = Roux-en-Y Gastric Bypass, SG = Sleeve Gastrectomy, TWL = Total weight loss

* Number of patients with genetic defects who underwent bariatric surgery.
** Genetic defects of varying pathogenicity.
